# Ability of Serum C-Reactive Protein Concentrations to Predict Complications After Laparoscopy-Assisted Gastrectomy: A Prospective Cohort Study

**DOI:** 10.1097/MD.0000000000003798

**Published:** 2016-05-27

**Authors:** Kecheng Zhang, Hongqing Xi, Xiaosong Wu, Jianxin Cui, Shibo Bian, Liangang Ma, Jiyang Li, Ning Wang, Bo Wei, Lin Chen

**Affiliations:** From the Department of General Surgery, Chinese People's Liberation Army General Hospital, Beijing, China.

## Abstract

Inflammatory markers, including C-reactive protein (CRP) and white blood cell (WBC), are widely available in clinical practice. However, their predictive roles for infectious complications following laparoscopy-assisted gastrectomy (LAG) have not been investigated. Our aim was to investigate the diagnostic accuracy of CRP concentrations and WBC counts for early detection of infectious complications following LAG and to construct a nomogram for clinical decision-making.

The clinical data of consecutive patients who underwent LAG with curative intent between December 2013 and March 2015 were prospectively collected. Postoperative complications were recorded according to the Clavien–Dindo classification. The diagnostic value of CRP concentrations and WBC counts was evaluated by area under the curve of receiver-operating characteristic curves. Optimal cutoff values were determined by Youden index. Univariate and multivariate logistic regression analyses were performed to identify risk factors for complications, after which a nomogram was constructed.

Twenty-nine of 278 patients (10.4%) who successfully underwent LAG developed major complications (grade ≥III). CRP concentration on postoperative day 3 (POD 3) and WBC count on POD 7 had the highest diagnostic accuracy for major complications with an area under the curve value of 0.86 (95% confidence interval [CI], 0.79–0.92] and 0.68 (95% CI, 0.56–0.79) respectively. An optimal cutoff value of 172.0 mg/L was identified for CRP, yielding a sensitivity of 0.79 (95% CI, 0.60–0.92) and specificity 0.74 (95% CI, 0.68–0.80). Multivariate analysis identified POD3 CRP concentrations ≥172.0 mg/L, Eastern Cooperative Oncology Group Performance Status ≥1, presence of preoperative comorbidity, and operation time ≥240 min as risk factors for major complications after LAG.

The optimal cut-off value of CRP on POD3 to predict complications following LAG was 172.0 mg/L and a CRP-based nomogram may contribute to early detection of complications after LAG.

## INTRODUCTION

Despite a global trend toward decreasing incidence, gastric cancer is highly prevalent in eastern Asia, especially in China.^1^ Surgery is the only curative treatment for gastric cancer. Postgastrectomy complication rates vary, ranging from 10.4% to 18.1% across studies,^2,3^ and usually have an unfavorable impact on long-time outcome in patients with gastric cancer.^4,5^ Hence, timely detection and management of postoperative infectious complications is of importance for both short- and long-term outcomes.

Markers of systemic inflammation, including C-reactive protein (CRP) and white blood cell (WBC), are widely available in clinical practice and are useful to identify patients at risk of infectious complications.^6–8^ With regard to gastric cancer, the study that has investigated the predictive value of these inflammatory markers for infectious complications is limited.^9,10^ Furthermore, these studies did not reach a consensus in terms of that what concentration of CRP or what postoperative day is suitable for detecting postoperative infectious complications. Conflicting findings from these results may be attributable to different operating procedures as it has been shown that patients who have undergone laparoscopic surgery have lower CRP concentrations than those who have undergone open procedures.^11,12^ Additionally, the retrospective design of previous studies may have contributed to this discrepancy.

In recent years, laparoscopy-assisted gastrectomy (LAG) has gained popularity and performed increasingly.^13^ To date, there is little evidence about the diagnostic role of inflammatory markers in predicting infectious complications following LAG. Therefore, the aim of the present study was to prospectively investigate the ability of systemic inflammation markers such as CRP concentration and WBC count to predict major postoperative complications in patients undergoing LAG, and then to construct a predictor-based nomogram for clinical use.

## METHODS

### Patient Data

This study was approved by the Chinese People's Liberation Army General Hospital Research Ethics Committee. Data of consecutive patients undergoing LAG for primary gastric cancer between December 2013 and March 2015 in the Chinese People's Liberation Army General Hospital were prospectively collected. Patients in whom conversion to open gastrectomy had been required were excluded. The following data were collected: age, sex, body mass index (BMI), performance status based on the Eastern Cooperative Oncology Group (ECOG) classification, physical status based on the American Society of Anesthesiologist classification, comorbidity, presence of neoadjuvant chemotherapy, relevant surgical variables (operation time, estimated blood loss, type of resection, extent of lymphadenectomy, type of reconstruction, presence of intraoperative transfusion), duration of hospitalization, postoperative complications according to the Clavien–Dindo classification,^14^ preoperative and postoperative CRP concentration and WBC count, tumor stage according to the third edition of the Japanese classification of gastric cancer,^15^ and tumor size.

### Determination of WBC and CRP Levels

Fasting blood was collected in 5 mL K2-EDTA Vacutainer (BD, Franklin Lakes, NJ) on each day morning. The WBC count was analyzed via automatic hematological blood analyzer (XE-2100, Sysmex Inc, China). Serum concentrations of CRP were measured by immune turbidimetric assay using automatic biochemical analyzer (BN-II, Siemens Healthcare Diagnostics, China). All the assays were performed at clinical laboratory of Chinese People's Liberation Army General Hospital according to manufacturer's protocol. The laboratory personnel were blinded to the clinical information.

### Perioperative Management

LAG was a standardized procedure performed by qualified surgeons with a minimal case load of 50 operations. All patients received preoperative antibiotic prophylaxis (500 mg metronidazole IV and 2000 mg ceftriaxone IV) 30 to 60 minutes before surgery. The extents of gastrectomy and lymphadenectomy were determined by the surgeons, who aimed to achieve R0 resection according to Japanese gastric cancer treatment guidelines.^15^ Anticoagulation was achieved with low molecular weight heparin adjusted for body weight and thromboembolic risk according to clinical history. A daily clinical assessment was performed postoperatively and additional examinations were performed as indicated clinically.

### Outcome Measures

The 30-day morbidity and mortality rates and duration of hospitalization were documented as outcome variables. Postoperative complications were graded according to the Clavien–Dindo classification and complications of grade III or more defined as major complications because they required surgical, endoscopic, or radiologic intervention.^14^ The following complications were analyzed: intra-abdominal fluid collection/abscess and pleural effusion confirmed by ultrasonography, computed tomography or drainage; anastomotic leakage and stump fistula confirmed by water-soluble contrast radiology; wound complications confirmed by purulent exudate with positive bacterial culture or requiring wound repair; pancreatic fistula confirmed by abdominal drainage with a serum amylase concentration greater than 3 times the upper limit after POD 3, and pneumonia confirmed by fever above 38.5°C with radiographic evidence and positive sputum culture.

### Statistical Analysis

Continuous variables are expressed as mean ± standard deviation (SD) or median with interquartile range (IQR) unless specified. The Mann–Whitney test or independent sample *t* test was used for continuous variables and the χ^2^ test for categorical variables. Diagnostic accuracy was determined by the area under the curve (AUC) of receiver-operating characteristics curves (ROC), and the 95% confidence intervals of the AUC were calculated according to Delong method.^16^ Youden index (sensitivity + specificity − 1) was applied to determine optimal cutoff values. Univariate and multivariate logistic regression analyses were used to identify clinical risk factors for major complication after LAG, after which a nomogram was constructed based on these risk factors. A 2-sided *P* value <0.05 was considered significant. SPSS statistical software version 17.0 (IBM SPSS, Chicago, IL) was used for all statistical analyses and the nomogram was constructed using R software version 3.2.2 with rms packages (http://www.r-project.org).

## RESULTS

### Patient Variables and Clinical Data

Between December 2013 and March 2015, 293 consecutive patients underwent LAG; however, conversion to an open procedure was required in 15 patients. After excluding these patients, the remaining 278 patients were included in the final analysis. Relevant patient characteristics are summarized in Table 1. The study cohort comprised 194 men and 84 women with a mean age of 59.4 ± 11.2 years. The mean BMI of the enrolled patients was 24.0 ± 3.3 kg/m^2^. Sixty-nine patients had comorbidities and 16 patients were given neoadjuvant chemotherapy. The mean operation time and median estimated blood loss were 216.2 ± 45.2 min and 150 (range 20–2300) mL, respectively. More than half of the patients (144/278) underwent D2 lymphadenectomy. The median duration of hospitalization was 18 (range 10–102) days. No patients died during their hospitalization. Twenty-nine patients (10.4%, 95% CI: 6.8–14.0%) developed major postoperative complications (grade III or greater according to the Clavien–Dindo classification), including 9 with anastomotic leakage, 6 with intra-abdominal abscess/fluid collections, 3 with pleural effusions, 4 with pneumonia, 3 with pancreatic fistulas, 2 with wound complications, and 2 with stump fistulas. The frequency of each type of complication and their onset times are summarized in Table 2.

**TABLE 1:**
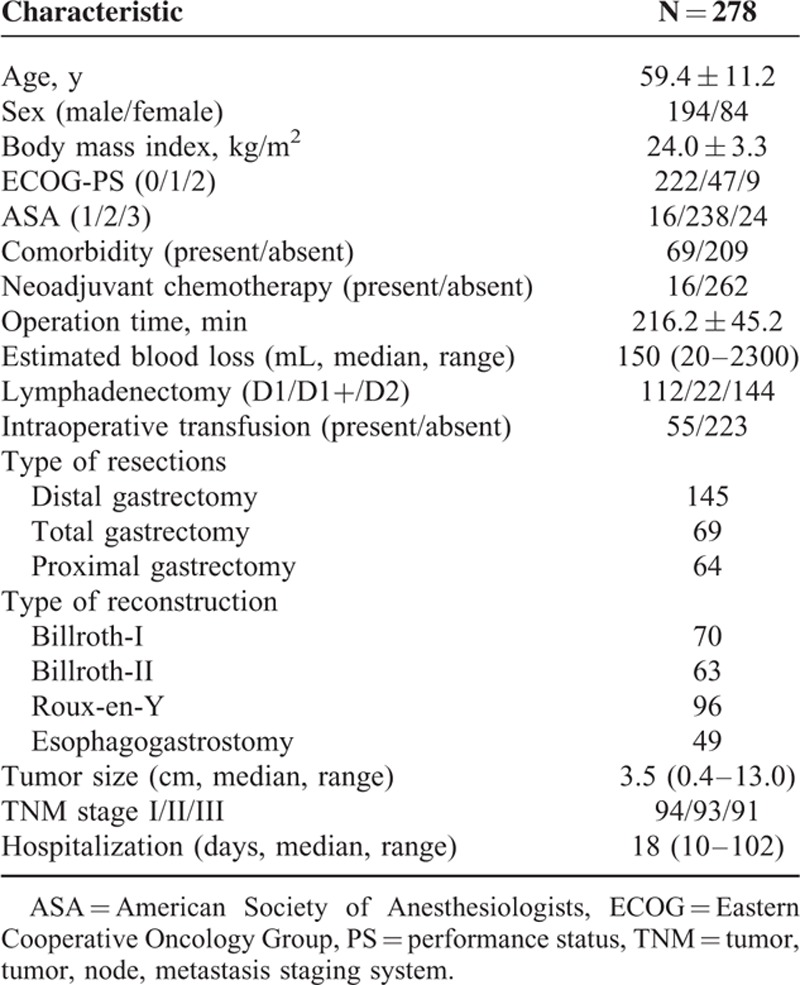
Participants’ Clinical and Surgical Variables

**TABLE 2:**
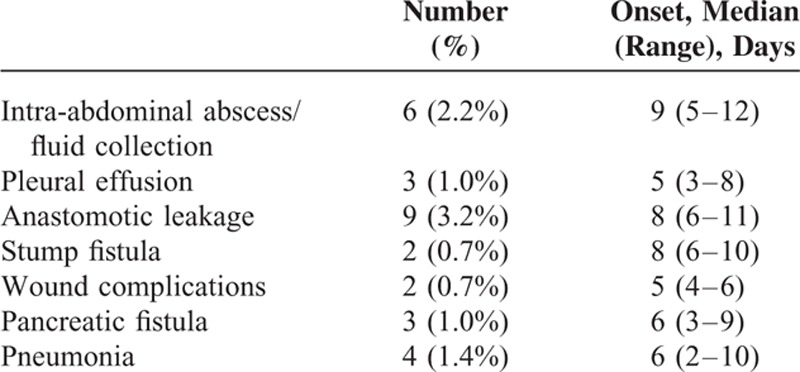
Frequency and Day of Onset of Each Complication

### Chronological Changes and Diagnostic Accuracy of CRP Concentration and WBC Count

**FIGURE 1:**
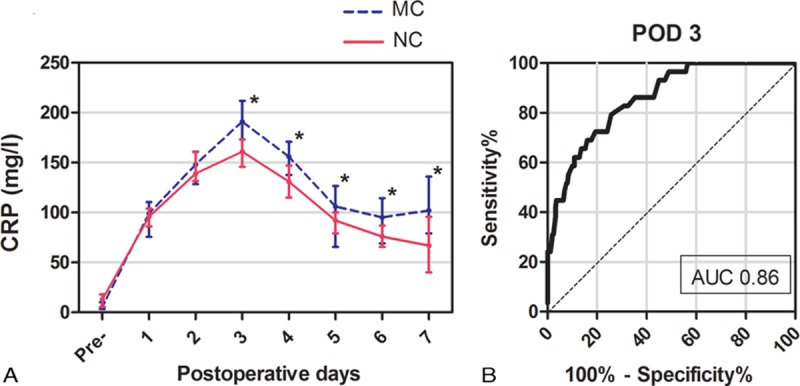
A, Serial changes in CRP concentrations (median with interquartile range) for major complications (MC) and no complications (NC). ^*∗*^*P* <0.05. B, Receiver-operating characteristic curve (ROC) for postoperative day 3 (POD3) CRP concentrations showing an AUC of 0.86 (95% CI, 0.79–0.92).

**FIGURE 2:**
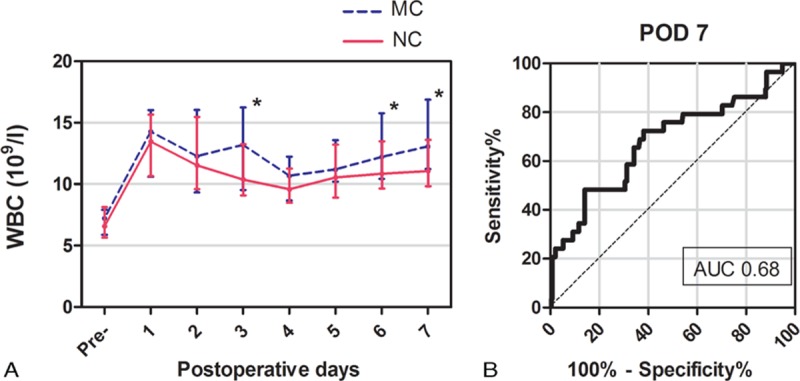
A, Serial changes in WBC counts (median with interquartile range) for major complications (MC) and no complications (NC). ^*∗*^*P* <0.05. B, Receiver-operating characteristic curve (ROC) for postoperative day 3 (POD3) WBC counts showing an AUC of 0.68 (95% CI, 0.56–0.79).

Perioperative changes in CRP concentration and WBC count are shown in Figures 1A and 2A, respectively. Similar changes in CRP concentrations were observed in patients with and without major complications. The CRP concentrations peaked on POD 3 and declined thereafter: from POD3 on there was a significant difference between patients with and without major complications. A sharp increase in WBC count was observed on POD 1, after which the WBC count fluctuated similarly in patients with and without major complications; however, patients who developed major complications had higher WBC counts. Both CRP concentrations and WBC counts changed significantly on POD 3. From POD 3 to POD 7, CRP concentrations were as follows: 174.0 (IQR, 156–195) mg/L versus 144.0 (IQR, 129.0–156.0) mg/L on POD 3 (*P* <0.001), 156.0 (IQR, 137.5–171.0) versus 131.0 (IQR, 115.0–147.0) mg/L (*P* <0.001) on POD 4, 106.0 (IQR, 65.5–126.5) mg/L versus 92.0 (IQR, 79.0–100.0) mg/L (*P* = 0.005) on POD 5, 95.0 (IQR, 69.0–114.5) mg/L versus 76.0 (IQR, 65.5–87.0) mg/L (*P* <0.001) on POD 6 and 102.0 (IQR, 79.0–136.0) mg/L versus 67.0 (IQR, 40.0–96.0) mg/L (*P* <0.001) on POD 7. For WBC count, these were 13.2 (IQR, 9.5–16.2) 10^9^/L versus 10.4 (IQR, 9.1–13.3) 10^9^/L (*P* = 0.001) on POD 3, 10.7 (IQR, 8.7–12.2) 10^9^/L versus 9.6 (IQR, 8.5–11.3) 10^9^/l (*P* = 0.256) on POD 4, 11.2 (IQR, 10.2–13.6) 10^9^/l versus 10.6 (IQR, 8.9–13.2) 10^9^/L (*P* = 0.190) on POD 5, 12.2 (IQR, 10.4–15.8) 10^9^/L versus 10.9 (IQR, 9.7–13.5) 10^9^/L (*P* = 0.004) on POD 6, and 13.1 (IQR, 11.2–16.9) 10^9^/L versus 11.1 (IQR, 9.8–13.6) 10^9^/L (*P* <0.001) on POD 7.

The diagnostic accuracy of CRP and WBC for predicting major complications was evaluated by the AUC. As shown in Figure 1B, the ROC curve of CRP concentration on POD 3 yielded a superior diagnostic accuracy with an AUC of 0.86 (95% CI, 0.79–0.92). The Youden index identified an optimal cutoff value of 172.0 mg/L with a sensitivity of 0.79 (95% CI, 0.60–0.92) and a specificity of 0.74 (95% CI, 0.68–0.80). However, regarding the predictive potential of WBC counts, ROC curve only yielded an AUC of 0.63 (95% CI, 0.51–0.76) on POD 3 and an AUC of 0.63 (95% CI, 0.52–0.74) on POD 6. As shown in Figure 2B, the superior value of the AUC was 0.68 (95% CI, 0.56–0.79) on POD 7 with a sensitivity of 0.69 (95% CI, 0.49–0.85) and a specificity of 0.63 (95% CI, 0.57–0.69).

### Risk Factors for Postoperative Major Complications and CRP-Based Nomogram

**FIGURE 3:**
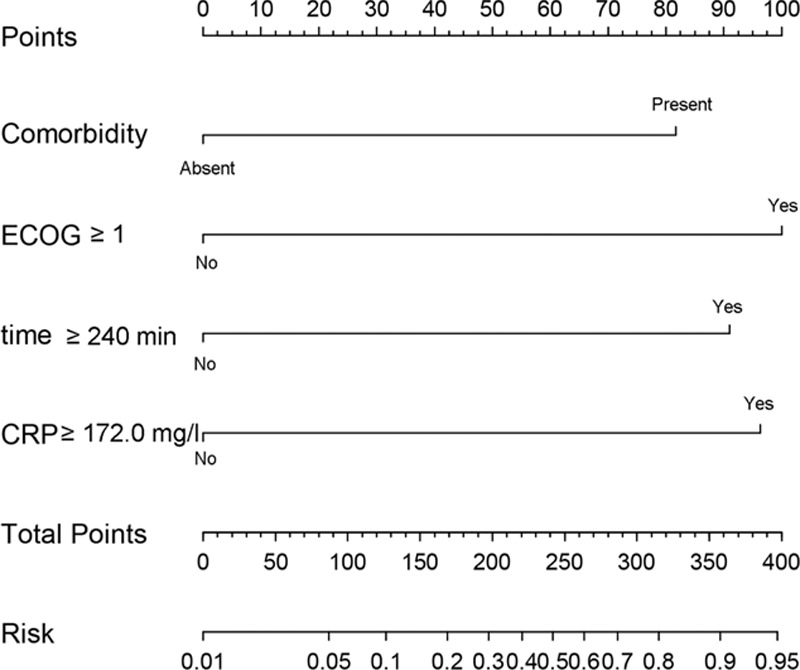
CRP-based nomogram for evaluation of an individual's risk of major complications following LAG.

The results of univariate and multivariate analysis are summarized in Table 3. It was found that POD3 CRP concentrations ≥172.0 mg/L, ECOG performance status ≥1, presence of preoperative comorbidity, and operation time ≥240 min were risk factors for major complications after LAG. Based on these risk factors, a nomogram was constructed for clinical evaluation of individual risk of major postoperative complications (Figure 3).

**TABLE 3:**
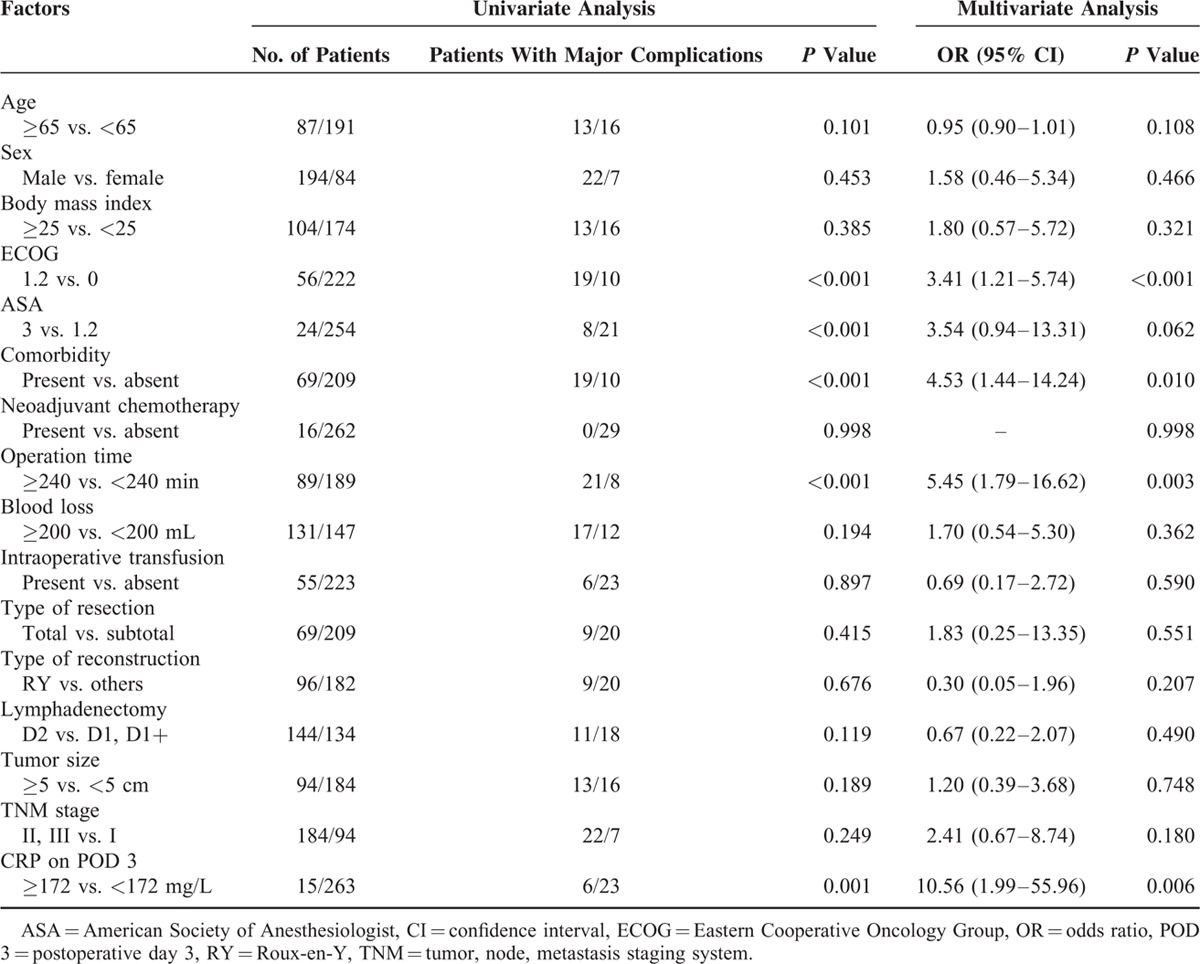
Significance of Selected Variables According to Univariate and Multivariate Analysis

## DISCUSSION

Recent studies have reported overall morbidity rates of 10.5% to 25.3% after LAG.^17–20^ Complications, particularly major ones such as anastomotic leakage and pancreatic fistula that require surgical or endoscopic interventions, pose threats to short- and long-term outcomes and result in longer hospital stays and higher medical expenses. Thus, early identification of patients at risk of complications is undoubtedly of great clinical value because it facilitates optimal timing of therapeutic interventions to minimize the sequelae of such complications.

CRP, a type of acute-phase protein, has a short and relatively constant half-life (19 h), is a reliable marker of acute inflammation, and has been investigated as a predictor of infectious complications after surgery.^6,7,9,10,21,22^ Previous studies have found that high CRP concentrations on POD 3 or POD 4 may contribute to predicting postoperative complications following abdominal surgery, reported AUC values ranging from 0.76 to 0.88.^6,8,21–23^ Three studies that have investigated the diagnostic potential of CRP concentrations for postoperative complications in patients with gastric cancer have reported differing results.^9,10,22^ Shishido et al reported that CRP on POD 3 had superior diagnostic accuracy for infectious complications with an AUC of 0.802 and an optimal cutoff value of 177 mg/L, whereas Warschkow et al reported that CRP had the maximal AUC value (0.77) on POD 4 with a cutoff value of 141 mg/L and Dutta et al concluded that CRP on POD 3 and POD 4 are both clinically useful for predicting complications with AUC values of 0.74 and 0.79, respectively.^9,10,22^ These relatively minor inconsistencies may be attributable to different sample sizes, bias caused by retrospective design, or different types of surgical procedures. Recently published articles have reported that laparoscopy-assisted surgery results in lower concentrations of CRP and proinflammatory cytokines than open surgery.^11,12,24^ Therefore, it may be important to investigate the diagnostic accuracy of CRP concentrations for complications after laparoscopy-assisted and open surgery separately.

In the present study, we investigated the ability of CRP concentrations to predict postoperative complications following LAG. The rate of major complications was 10.4% (95% CI: 6.8–14.0%), which is slightly higher than recently reported rates of 5.1% to 8.3%.^3,25^ One possible explanation for this discrepancy is the higher tumor stage and extent of lymphadenectomy in our study than in those cited. Another is that a greater proportion of cases underwent total gastrectomy in our study: the complication rate following laparoscopy-assisted total gastrectomy is higher than for partial gastrectomy.^25,26^ As illustrated in Figure 1, CRP concentration on POD 3 had optimal diagnostic accuracy for predicting postoperative complications with an AUC of 0.86 (95% CI, 0.79–0.92), sensitivity of 0.79 (95% CI, 0.60–0.92), and specificity of 0.74 (95% CI, 0.68–0.80), which are consistent with previous studies.^6,9,10,27^ According to the suggested guidelines for interpretation of AUC values, CRP on POD 3 had moderate (0.7 < AUC < 0.9) ability to predict postoperative complications.^28^ We identified an optimal cutoff value of 172.0 mg/L in this study, which is lower than the cutoff values of 177.0 and 180.0 mg/L cited by Shishido et al and Dutta et al.^9,22^ This result corresponds well to the conclusion that cutoff values are lower for laparoscopic than for open procedures.^12^ As shown in Table 2, the median day of onset of complications ranged from POD 5 to POD 9, suggesting that CRP concentration on POD 3 would facilitate early detection of complications before the development of obviously clinical symptoms. Early detection can result in early interventional treatment, which might improve patients’ short-term outcomes.

Serial changes in postoperative WBC counts (Figure 2) were significantly different on POD 3, 6, and 7. However, only WBC counts on POD 7 had superior diagnostic accuracy for predicting postoperative complications, the AUC being only 0.68 (95% CI, 0.56–0.79), indicating low predictive ability (0.5 < AUC < 0.7) according to Swets study.^28^ Additionally, by POD 7 most postoperative complications have obvious clinical symptoms. Thus, postoperative WBC counts do not predict complications accurately or in a timely manner. Our results are consistent with previous studies that showed that CRP concentrations are more useful than WBC counts for predicting postoperative complications.^8,22,29^ Other promising markers such as procalcitonin and interleukin-6 have recently been reported as predictors of infection after major abdominal surgery.^30^ However, use of these markers is controversial and more expensive than measurement of CRP concentrations.^31,32^ We did not measure procalcitonin and interleukin-6 concentrations because they are not well-established indicators and would have added to patient's medical costs.

Unlike previous studies, we also identified risk factors for postoperative complications (Table 3) and used these risk factors to construct, to the best of our knowledge, the first CRP-based nomogram for evaluation of individual risk of postoperative major complications (Figure 3). Nomograms are widely used in oncology and medicine. With their ability to generate an individual probability of a clinical event by integrating risk factors, user-friendly interfaces, and easily understood calculations and interpretation, nomograms could facilitate more personalized medical practice.^33,34^ For example, as shown in Figure 3, a patient with comorbidity, ECOG ≥ 1, operation time ≥240 min, and POD 3 CRP ≥172.0 mg/L would score 82, 100, 90, and 97 points, respectively. The total of 369 points corresponds to a risk of 0.92; that is, this patient has a 92% chance of developing major postoperative complications and should be managed accordingly.

Our results confirmed that changes of CRP concentrations postoperatively were significantly correlated with the development of infectious complications, whereas changes of WBC count were not. We have also investigated whether high concentration CRP were associated with clinic-pathological variables such as TNM stage and lymph node involvement; however, no significant association was observed (data not shown). A rise in CPR concentrations is usually considered the result, rather than a cause, of infectious complications. CRP was seemed to be more sensitive to the presence of infection. In fact, CRP functions as an early defense against infection in innate immunity, facilitating complement-binding to foreign and damaged cells and enhancing phagocytosis by macrophages. CRP has not only shown to provide a link between the innate and adaptive immunity, but also shown to correlate T-lymphocyte function, stress response, and the degree of hyperglycemia,^35^ which were important factors associated with the promotion of bacterial growth and the development of postoperative infectious complications.^36,37^ Therefore, in addition to its predictive value for a clinical infection, CRP may be involved in the modulation of postoperative immune function of patients with gastric cancer.

In conclusion, the present study identified that CRP concentration on POD 3 with a cutoff value of 172.0 mg/L would be useful for predicting postoperative major complications following LAG. The CRP-based nomogram we have constructed from our data could usefully contribute to clinical decision-making.
